# Complete Genome Sequence of *Salmonella enterica* subsp. *enterica* Serovar Typhi Isolate B/SF/13/03/195 Associated with a Typhoid Carrier in Pasir Mas, Kelantan, Malaysia

**DOI:** 10.1128/genomeA.01285-15

**Published:** 2015-11-12

**Authors:** Salwani Muhamad Harish, Kee-Shin Sim, Fauziah Mohd Nor, Hani Mat Hussin, Wan Mansor Hamzah, Nazalan Najimudin, Ismail Aziah

**Affiliations:** a Institute for Research in Molecular Medicine (INFORMM), Health Campus, Universiti Sains Malaysia, Kubang Kerian, Kelantan, Malaysia; b School of Biological Science, Main Campus, Universiti Sains Malaysia, Pulau Pinang, Malaysia; c CDC Unit, Kelantan State Health Department, Kota Bharu, Kelantan, Malaysia; d Kota Bharu Public Health Laboratory, Jalan Kuala Krai, Kota Bharu, Kelantan, Malaysia; e Public Health Department, Kelantan State Health Department, Wisma Persekutuan, Kota Bharu, Kelantan, Malaysia

## Abstract

We report here the complete genome sequence of *Salmonella enterica* subsp. *enterica* serovar Typhi B/SF/13/03/195 obtained from a typhoid carrier, who is a food handler in Pasir Mas, Kelantan.

## GENOME ANNOUNCEMENT

*Salmonella enterica* subsp. *enterica* serovar Typhi is a human-restricted pathogen that causes typhoid fever (1, 2). The high incidence of typhoid fever in developing countries is due to the persistence and survival of the organism in the host, establishing an asymptomatic chronic carrier (3–5). Typhoid fever is common in areas where public water supplies are contaminated and that have improper sewage disposal (4). It can also be transmitted through contaminated food and water by food handlers who are carriers (6).

*Salmonella enterica* subsp. *enterica* serovar Typhi B/SF/13/03/195 was isolated from stool samples from a typhoid carrier who is a food handler in Pasir Mas, Kelantan, Malaysia, in 2013. The isolate was confirmed using a culture method, serological test, and PCR (7). The genome of the isolate was sequenced by Pacific Biosciences (PacBio) RS II single-pass sequencing with P4-C_2_ chemistry and a 10-kb library for a 180-min collection sequencing protocol (Pacific Biosciences, Menlo Park, CA). The 10-kb continuous long read (CLR) was *de novo* assembled using the PacBio Hierarchical Genome Assembly Process (HGAP), followed by Minimus 2, and was polished by Quiver.

A single contig of 4,799,139 bp was generated from the assembly with 52% G+C content. The genome did not harbor any plasmid. The gene prediction was performed by Glimmer3 (8) and RAST (9), and the functional annotation was done by Blast2GO (10), which predicted 4,945 genes for protein-coding sequences (CDSs), with an average length of 848 bp, 22 rRNA genes (11), and 80 tRNA genes (12).

Based on a whole-genome investigation, the genome sequence was found to encode nitrate reductase and sigma factor RpoS, as previously reported in two published genomes, those of CT18 and Ty2 (13). Sigma factor RpoS is reported to be involved in resistance to various types of environmental stresses, including starvation, oxidation, and low pH, while nitrate reductase plays an important role in the regulation of anaerobic growth (14, 15). The genome sequence also revealed several pathogenicity islands and hypothetical proteins.

### Nucleotide sequence accession number.

The genome sequences have been deposited in GenBank under the accession no. CP012151.
